# MicroRNA-16 Modulates HuR Regulation of Cyclin E1 in Breast Cancer Cells

**DOI:** 10.3390/ijms16047112

**Published:** 2015-03-30

**Authors:** Xun Guo, Melanie C. Connick, Jennifer Vanderhoof, Mohammad-Ali Ishak, Rebecca S. Hartley

**Affiliations:** Department of Cell Biology and Physiology, University of New Mexico Health Sciences Center, Albuquerque, NM 87131, USA; E-Mails: xunguo1128@gmail.com (X.G.); mconnick@unm.edu (M.C.C.); jennmv1@icloud.com (J.V.); mishak@salud.unm.edu (M.-A.I.)

**Keywords:** cyclin E1, miR-16, HuR, breast cancer cells, post-transcriptional regulation

## Abstract

RNA binding protein (RBPs) and microRNAs (miRNAs or miRs) are post-transcriptional regulators of gene expression that are implicated in development of cancers. Although their individual roles have been studied, the crosstalk between RBPs and miRNAs is under intense investigation. Here, we show that in breast cancer cells, cyclin E1 upregulation by the RBP HuR is through specific binding to regions in the cyclin E1 mRNA 3' untranslated region (3'UTR) containing U-rich elements. Similarly, miR-16 represses cyclin E1, dependent on its cognate binding sites in the cyclin E1 3'UTR. Evidence in the literature indicates that HuR can regulate miRNA expression and recruit or dissociate RNA-induced silencing complexes (RISC). Despite this, miR-16 and HuR do not affect the other’s expression level or binding to the cyclin E1 3'UTR. While HuR overexpression partially blocks miR-16 repression of a reporter mRNA containing the cyclin E1 3'UTR, it does not block miR-16 repression of endogenous cyclin E1 mRNA. In contrast, miR-16 blocks HuR-mediated upregulation of cyclin E1. Overall our results suggest that miR-16 can override HuR upregulation of cyclin E1 without affecting HuR expression or association with the cyclin E1 mRNA.

## 1. Introduction

Cyclin E1 is expressed briefly during the G1−S transition of the cell division cycle when it binds to and activates cyclin-dependent kinase 2 (Cdk2). Cyclin E1/Cdk2 complexes promote initiation of DNA replication and centrosome duplication, after which cyclin E1 is phosphorylated and degraded by ubiquitin-mediated proteolysis, inactivating Cdk2 and allowing normal cell cycle progression [1,2,3,4,5]. While a predominant function of cyclin E1 is to activate Cdk2, there are also Cdk-independent functions including formation of pre-replication complexes on DNA, endocycling, centrosome duplication, re-entry into the cell cycle from G0, cellular transformation, and stem cell maintenance [6,7,8]. Truncated variants of cyclin E1, termed low molecular weight (LMW) isoforms are able to induce malignant transformation [9], and oncogenesis has been associated with increased cyclin E1 in the absence of increased Cdk2 activity [9,10,11].

Experimental and pathologic evidence indicate that cyclin E1 deregulation is oncogenic. Cyclin E1 is overexpressed and present throughout the cancer cell cycle [12] where it advances S phase entry [13] and induces genetic instability [14]. Ectopic expression in mammary epithelium of transgenic mice leads to hyperplasia and carcinoma [15], while its reduction suppresses tumor development in a mouse breast cancer model [16]. Elevated cyclin E1 is associated with aggressive disease in a variety of human tumors including breast, for which it is one of the most reliable independent prognostic markers [17,18]. In breast cancer, cyclin E1 is overexpressed in 30% of patients, including overexpression of both the full length (50 kDa) and several LMW isoforms (ranging in size from 33 to 45 kDa) [17,19].

Cyclin E1 overexpression in breast cancer is associated with increased tumor grade, estrogen receptor (ER) negative status, progesterone receptor negative status, and proliferative index by Ki67 staining [20,21]. Cyclin E1 expression has an inverse linear relationship with metastasis free survival [22], and high expression of cyclin E1 mRNA also predicts poor overall survival [21], shorter metastasis-free survival in both ER negative and ER positive patients, and a shorter relapse-free interval, including tamoxifen treated patients [20].

Disruptions in both transcriptional and post-translational regulation result in cyclin E1 overexpression in cancer. Similar to other forms of cyclin E1 deregulation, post-transcriptional regulation of cyclin E1 is also disrupted in cancer. Increased mRNA stability results in cyclin E1 overabundance during all phases of the cell cycle [23]. RNA binding proteins (RBPs) and microRNAs (miRNAs or miRs) regulate cyclin E1 mRNA stability. The RBP HuR is overexpressed in primary breast tumors [24,25,26,27] and breast cancer cells, where it stabilizes cyclin E1 mRNA, leading to cyclin E1 protein overexpression [28]. Cyclin E1 mRNA is also targeted by miR-15b and miR-16 [29,30], both of which destabilize the mRNA and decrease cyclin E1 protein. These miRNAs are decreased in breast cancer [31,32]. Since both HuR and miRNAs regulate cyclin E1 and both are deregulated in breast cancer, we set out to determine if HuR and miR-16 cooperate to control cyclin E1 level in breast cancer cells.

## 2. Results and Discussion

### 2.1. Both HuR and miR-16 Regulate Cyclin E1 in Breast Cancer Cells

**Figure 1:**
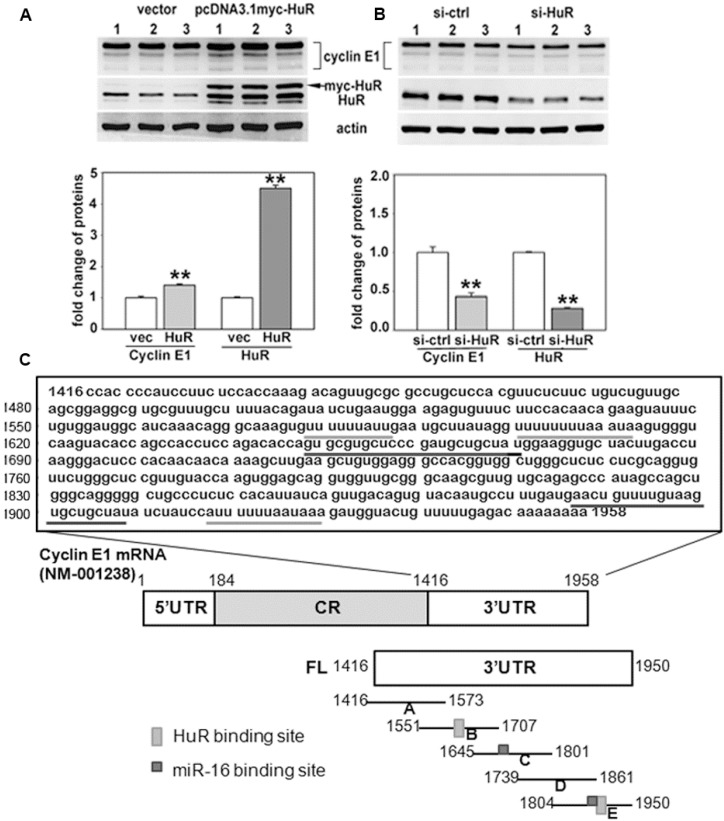

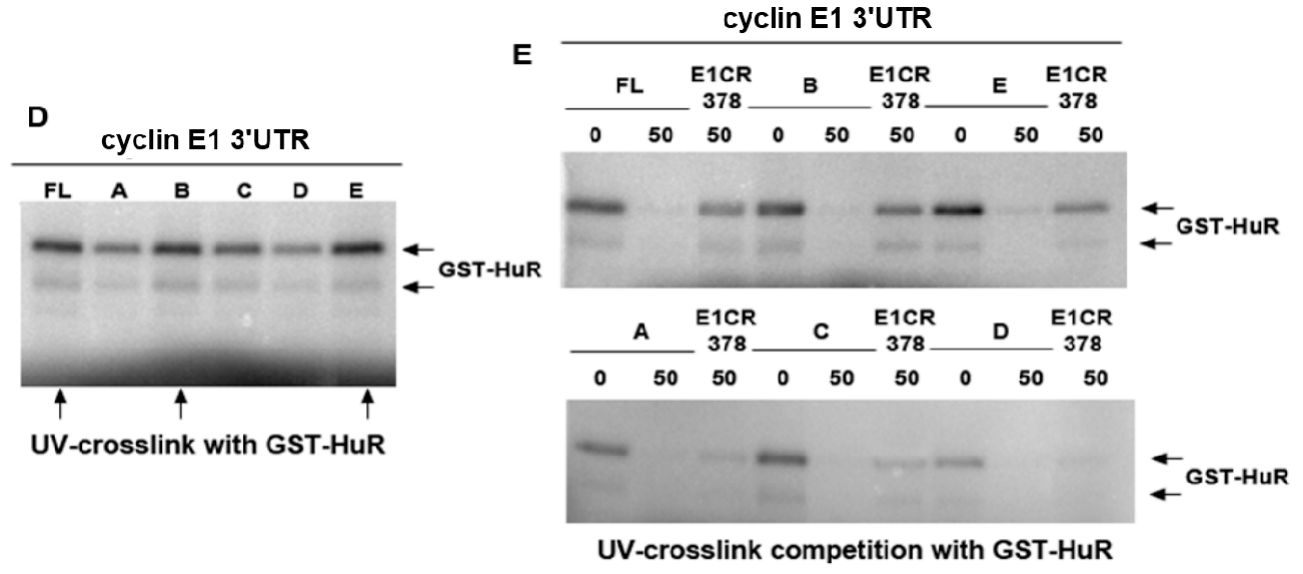
HuR binds U-rich regions of the cyclin E1 3'UTR. (**A**) MCF7 cells were transfected with pcDNA3.1 (vec) or pcDNA3.1 myc-HuR (HuR) or (**B**), with control siRNA (si-ctrl) or HuR siRNA (si-HuR). 48–72 h after transfection, protein was extracted for western blotting for Cyclin E1 or HuR (top). Values in the graphs are the mean fold change ± SD from three independent experiments. ** *p* < 0.01 *versus* control; (**C**) Sequence of cyclin E1 3'UTR with two HuR binding sites (underlined with light grey) and two miR-16 target sites (underlined with dark grey), as well as a schematic depiction of the cyclin E1 mRNA including the 5'UTR, coding region (CR) and 3'UTR. FL (full length cyclin E1 3'UTR) and (**A**–**E**) are 3'UTR segments used for UV cross-link and UV cross-link competition experiments shown in panels D and E; (**D**) The binding of GST-HuR with the cyclin E1 3'UTR detected by UV cross-link analysis; and (**E**) Specific or non-specific binding of GST-HuR with the cyclin E1 3'UTR measured by UV cross-link competition using E1CR378 (bases 184–378 in the coding region) as non-specific competitor. Representative crosslinks of at least three replicates are shown in panels D and E.

We previously showed that HuR binds to and stabilizes cyclin E1 mRNA, thereby contributing to cyclin E1 overexpression in breast cancer cells [28]. Confirming this, overexpression of HuR increased cyclin E1 protein 1.5 fold in MCF-7 breast adenocarcinoma cells (Figure 1A), while HuR knockdown reduced cyclin E1 by half (Figure 1B). HuR binds AU-rich and U-rich elements as well as polypyrimidine tract motifs, primarily in introns and the 3' untranslated regions of mRNAs [33,34]. HuR binding motifs in the cyclin E1 3'UTR were identified using PAR-CLIP [34], with 2-4 binding sites predicted. In order to verify the HuR binding site(s) in the cyclin E1 mRNA (Genbank NM001238), site-directed mutagenesis was used to create RNAs consisting of overlapping regions of the 3'UTR each spanning approximately 150 nucleotides (Figure 1C, regions A–E). UV cross-link experiments with *in vitro* transcribed, radiolabeled RNA and GST-HuR revealed that, as predicted, HuR bound 3'UTR regions containing U-rich elements (Figure 1D, regions B and E) similarly to the full length 3'UTR (FL). It also bound 3'UTR regions without U-rich elements (A, C and D), but less well. Since HuR bound all regions, we performed UV cross-link competition assays to determine which regions were bound specifically. Figure 1E shows that HuR binding to region B (nucleotides 1551–1707) and region E (nucleotides 1804–1950) was competed by nonradiolabeled full length cyclin E1 3'UTR (FL), but not by a partial cyclin E1 coding region (E1CR378), while HuR binding to regions A, C, and D was efficiently competed by both the cyclin E1 3'UTR and E1CR378. We conclude that HuR specifically binds U-rich regions B and E of the cyclin E1 3'UTR. These regions contain RNA recognition element 1 (RRE1, UUUUUA) and RRE3 (AUUUU) [34] and poly(U), a previously known HuR motif that was also identified by the more recent PAR-CLIP studies [33,34].

**Figure 2:**
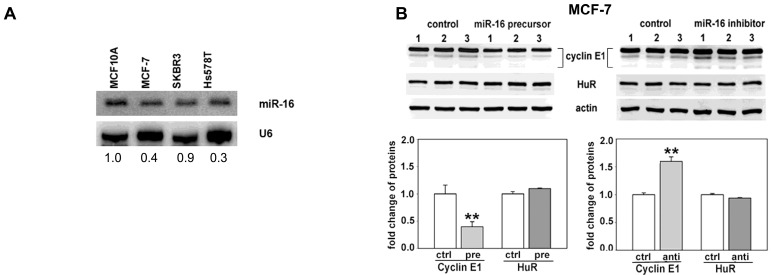

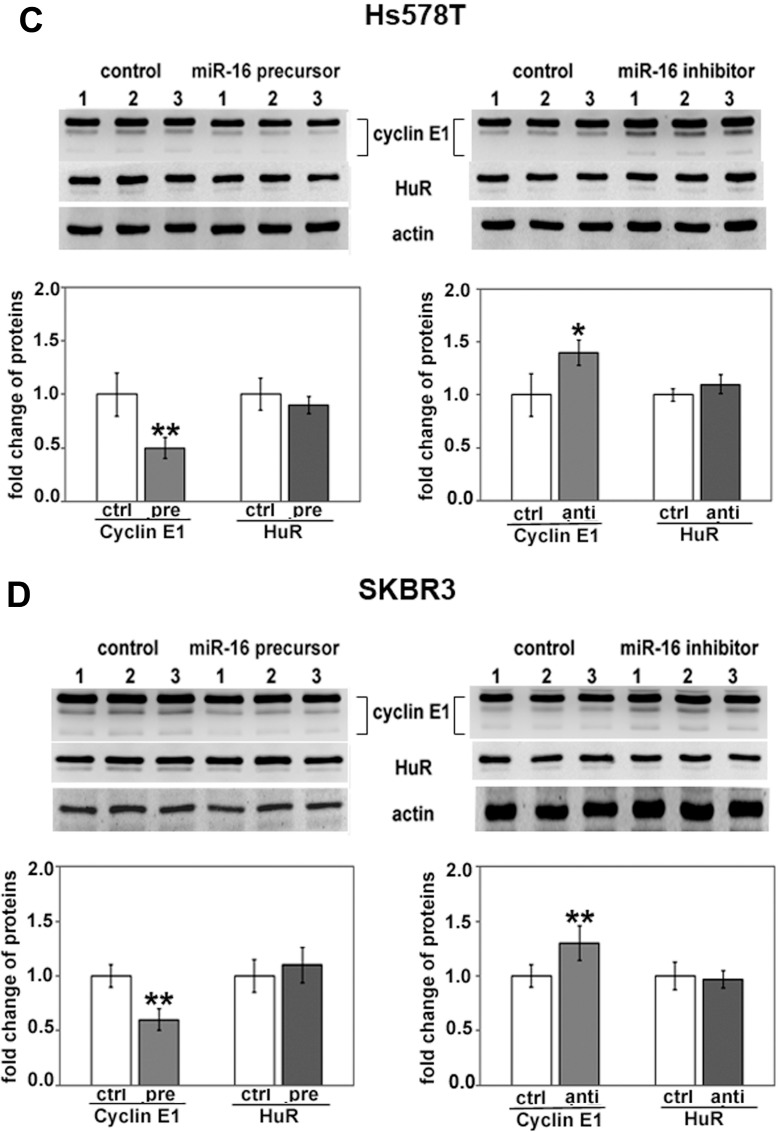
miR-16 regulates cyclin E1 in breast cancer cells. (**A**) Northern analysis of miR-16 level in a nontumorigenic breast epithelial cell line (MCF10A) and three different breast cancer cell lines (MCF7, SKBR3, and Hs578T). Blot was reprobed for U6 snRNA. Bottom numbers are density relative to MCF10A cells after normalization to U6 snRNA; (**B**–**D**) The indicated breast cancer cell lines were transfected with control miRNA (ctrl), miR-16 precursor (pre) or antagomir (anti). The level of cyclin E1, HuR and loading control actin were assessed by western blot analysis (**top**). Numbers 1–3 represent three separate experiments. Cyclin E1 and HuR western blot signals were quantified by densitometry (**bottom**). Values are the means ± SD from three independent experiments. * *p* < 0.05 *versus* ctrl; ** *p* < 0.01 *versus* ctrl.

**Figure 3:**
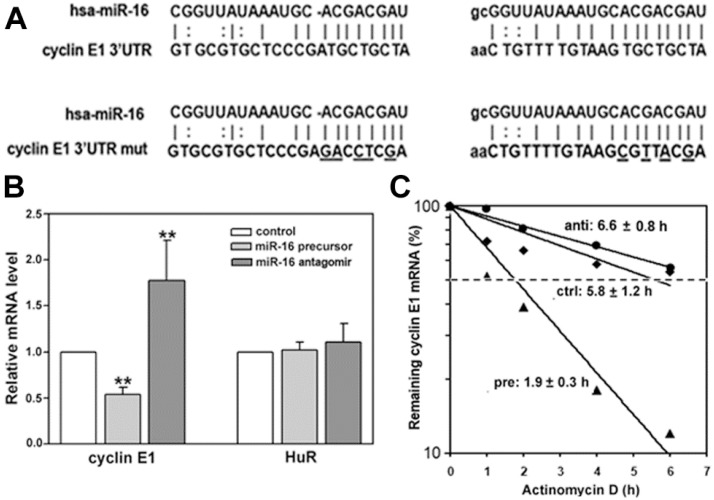

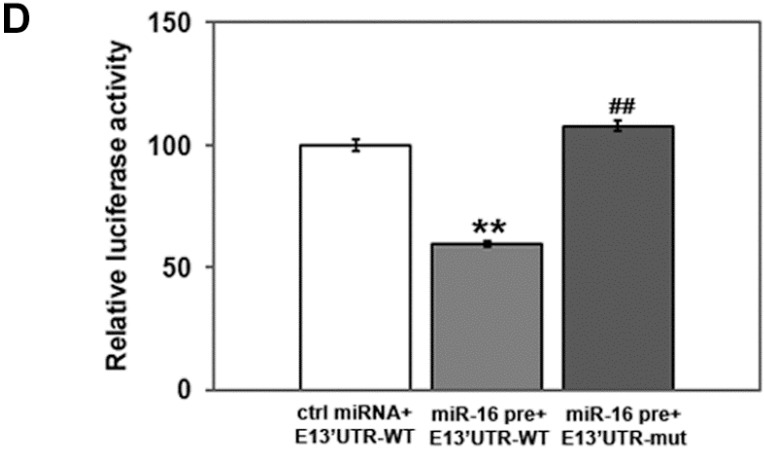
miR-16 destabilizes cyclin E1 mRNA via binding its 3'UTR. (**A**) hsa-miR-16 sequence and its target sequences in the cyclin E1 3'UTR (**top**), or (**bottom**) the cyclin E1 3'UTR with mutations in the miR-16 seed sequences (cyclin E1 3'UTR mut; changed bases are underlined). After a 48 h transfection of MCF7 cells with miR-16 precursor or antagomir; (**B**) cyclin E1 and HuR mRNA levels were assessed by qRT-PCR; (**C**) Cyclin E1 mRNA stability was assessed by qRT-PCR after addition of actinomycin D. Data were normalized to GAPDH and expressed as relative mRNA levels. Numbers indicate the mRNA half-life in hours (h) in cells treated with control miR (ctrl), miR-16 precursor (pre) or miR-16 antagomir (anti); and (**D**) MCF-7 Cells were co-transfected with control miRNA (ctrl miRNA) or miR-16 precursor (miR-16 pre), pMIR-REPORT β-gal control vector and pMIR-REPORT luciferase vector containing either the cyclin E1 3'UTR (E13'UTR-WT) or the cyclin E1 3'UTR with miR-16 seed sequence mutations (E13'UTR-mut, Figure 2a). Cell extracts were prepared 24 h after transfection, and luciferase activity measured using the Dual-Light System. The relative luciferase activity after normalization to pMIR-REPORT β-gal control plasmid is shown. *n* = 4, ** *p* < 0.01 *versus* ctrl miRNA; ^##^ *p* < 0.01 *versus* miR-16 pre+ E13'UTR-WT.

In addition to the U-rich elements in regions B and E, two predicted miR-16 target sites are contained in regions C and E of the cyclin E1 3'UTR (nucleotides 1649–1671, and 1887–1909, Figure 1C and Figure 3A). The proximity of these binding sites to the AREs, especially in region E, suggested the possibility that HuR and miR-16 could affect the other’s binding and thus regulation of cyclin E1 mRNA. Before exploring this possibility, we first confirmed that miR-16 is decreased in different breast cancer cell lines. Figure 2A shows that miR-16 is downregulated in MCF-7 and Hs578T breast cancer cell lines as compared to non-tumorigenic MCF10A breast epithelial cells. These cell lines represent different breast cancer subtypes. MCF-7 cells are ER^+^PR^+^Her2^−^, Luminal; Hs578T cells are ER^−^PR^−^Her2^−^, Basal B; and SKBR3 cells are ER^−^PR^−^ERBB2^+^, Luminal. Regardless of receptor status or subtype, introducing miR-16 precursor decreased cyclin E1 protein while miR-16 antagomir increased cyclin E1 protein in these breast cancer cell lines (Figure 2B–D, triplicate experiments are shown) as well as in MCF10A cells (data not shown).

As miR-16 targets HuR itself [35], we also assessed HuR protein level in miR-16 altered cells. HuR level did not change in response to miR-16 alteration in any of the cell lines assessed (Figure 2B–D). Collectively, these data show that cyclin E1 is regulated by miR-16 without affecting HuR level. miR-16 likely targets cyclin E1 directly, with its reduction directly contributing to overexpression of cyclin E1 in these cells.

### 2.2. miR-16 Represses Cyclin E1 Dependent on Cognate Binding Sites within the 3'UTR of Its mRNA

We next asked how miR-16 regulated expression of cyclin E1. In general, miRNAs control gene expression by targeting mRNAs for either translational repression or degradation. To assess the mechanism, we first performed qRT-PCR using MCF-7 cells to determine if the cyclin E1 mRNA level was altered after introducing miR-16 precursor or antagomir. Cyclin E1 mRNA level was significantly altered (Figure 3B), decreasing upon anti-miR-16 treatment, and increasing after pre-miR-16 treatment. HuR mRNA level was not affected by altering miR-16 (Figure 3B). We next asked if these effects were due to altering cyclin E1 mRNA half-life (Figure 3C). Cyclin E1 mRNA half-life was only slightly increased by antagomiR-16 (from 5.8 to 6.6 h), as would be expected given the already reduced level of miR-16 in MCF-7 cells. In contrast, pre-miR-16 decreased the half-life of cyclin E1 mRNA from 5.8 to 1.9 h.

To determine if regulation of cyclin E1 mRNA by miR-16 was direct, we mutated the miR-16 seed sequences in the cyclin E1 3'UTR (Figure 3A) and assessed effects on a luciferase reporter containing either the mutated or wild-type 3'UTR (Figure 3D). miR-16 precursor reduced the activity of a luciferase reporter fused to the wild-type cyclin E1 3'UTR by 40% (Figure 3D, E13'UTR-WT). Four or five point mutations in the cyclin E1 3'UTR complementary to the miR-16 seed sequence (Figure 3A) completely restored the luciferase activity of pre-miR-16 transfected cells (Figure 3D, E13'UTR-mut). These results indicate that miR-16 represses cyclin E1 dependent on miR-16 cognate binding sites within the 3'UTR of its mRNA.

### 2.3. HuR Does not Affect miR-16 Expression nor Do HuR and miR-16 Affect the Other’s Binding

**Figure 4:**
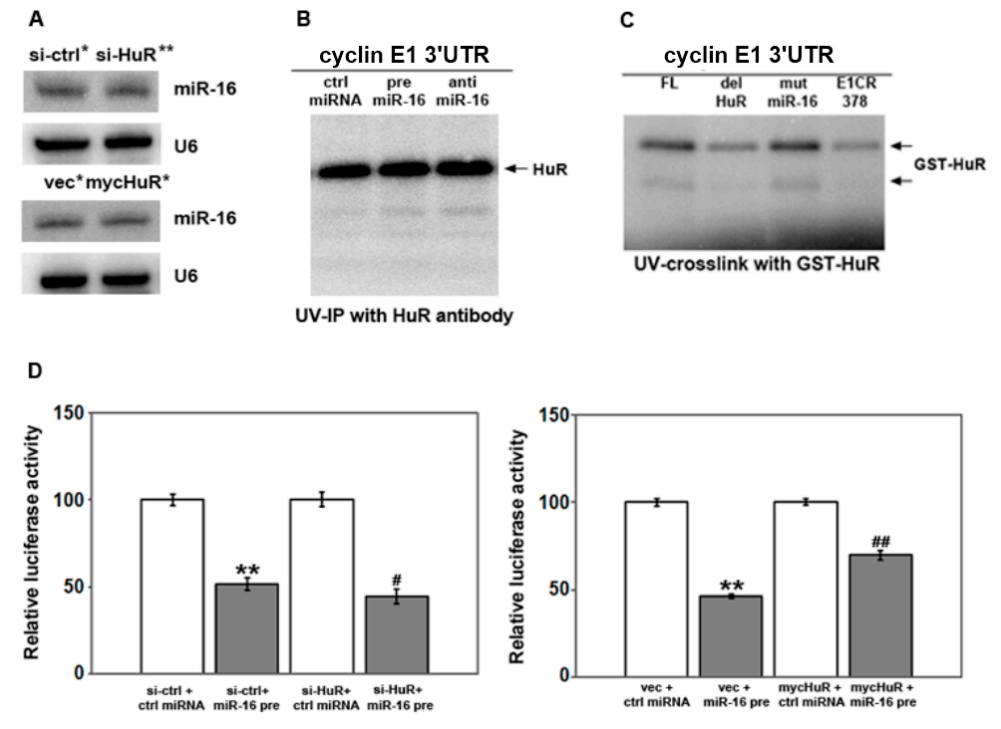
The affect of miR-16 on a reporter mRNA depends on HuR level. (**A**) MCF-7 cells were transfected with control siRNA or si-HuR, pcDNA3.1 vector or pcDNA3.1mycHuR for 48 h. miR-16 level was assessed by Northern blotting. The blots were reprobed with U6 snRNA as an internal control. Relative density was calculated relative to either si-ctrl or vec after normalization to U6 snRNA: * density = 1, ** density = 0.9; (**B**) 48 h after transfection of miR-16 precursor (pre miR-16) or antagomir (anti miR-16), the binding of HuR to the cyclin E1 3'UTR was detected by UV-crosslink immunoprecipitation. A representative experiment of three is shown; (**C**) UV-crosslink of GST-HuR with the full length cyclin E1 3'UTR (FL), the cyclin E1 3'UTR with the HuR binding sites deleted (del HuR) or the miR-16 sites deleted (mut miR-16), or the cyclin E1 coding region (E1CR378); (**D**) Cells were co-transfected with control miRNA or miR-16 precursor, pMIR-REPORT β-gal control vector or pMIR-REPORT luciferase vector containing either the cyclin E1 3'UTR 48 h after transfection with control siRNA or si-HuR, vector or pcDNA3.1mycHuR. Luciferase activity was measured using the Dual-Light System. The relative luciferase activity after normalization to pMIR-REPORT β-gal control plasmid is shown. The experiments were repeated three times. ** *p* < 0.01 *versus* si-ctrl or vec plus ctrl miRNA; ^#^ *p* < 0.05 *versus* si-ctrl plus miR-16 precursor; ^##^ *p* < 0.01 *versus* vec plus miR-16 precursor.

Cyclin E1 is regulated by both HuR and miR-16, and miRNAs and HuR have been shown to regulate the other’s function in a variety of ways [36]. HuR was reported to destabilize miR-16 in colon cancer cells by an unknown mechanism [37], as well as to potentially decrease miR-7 by decreasing its processing [33]. Since miR-16 is decreased in breast cancer cells overexpressing HuR (Figure 2B and [38]), one possibility is that HuR decreases miR-16 in these cells. However, knockdown or overexpression of HuR had no effect on miR-16 level as shown by Northern analysis (Figure 4A) or by qRT-PCR (not shown).

HuR has been shown to competitively regulate mRNAs with miRNAs [36,37,39,40,41], as well as to cooperate with miRNAs to regulate target mRNAs [42,43,44]. To determine if HuR and miR-16 could compete or cooperate to bind the cyclin E1 mRNA, we performed UV-crosslink analysis with radiolabeled cyclin E1 3'UTR and cells treated with pre-miR-16 and anti-miR-16, followed by immunoprecipitation of HuR. (Figure 4B). HuR binding to the cyclin E1 3'UTR was not affected by increasing miR-16 (compare ctrl miRNA to pre-miR-16), or by antagonizing its function (anti-miR-16). Next, we mutated the miR-16 target sites in the cyclin E1 3'UTR and assessed binding of GST-HuR. Figure 4C shows that mutation of the miR-16 sites (see Figure 2A for mutations) did not affect HuR binding to the cyclin E1 3'UTR, while deletion of the HuR binding sites decreased HuR binding as compared to the full length 3'UTR (FL). Table 1 lists the primers used to delete the U-rich regions indicated by grey underlining in Figure 1C at nucleotides 1578-1586 and 1600-1612 (these were deleted together, HuR site 1), and 1917–1929 (HuR site 2). We conclude that miR-16 does not appear to interfere with or enhance HuR binding to the cyclin E1 3'UTR.

### 2.4. HuR Partially Blocks miR-16 Repression of a Reporter mRNA Containing the Cyclin E1 3'UTR

To determine if HuR and miR-16 could affect the other’s function, we assessed activity of the luciferase-cyclin E1 3'UTR reporter in cells with altered levels of HuR, miR-16 or both (Figure 4D). Consistent with results in Figure 3C that showed that pre-miR-16 decreased cyclin E1 mRNA level, miR-16 precursor reduced luciferase activity by 50% in cells pre-treated with control siRNA as compared to cells treated with control miRNA and control siRNA (Figure 4D, left panel). Pre-treatment with si-HuR reduced luciferase activity a further 10% (si-HuR + miR-16 pre). Conversely, increasing HuR level by overexpressing myc-tagged HuR prior to introducing pre-miR-16 (Figure 4D, right panel, mycHuR + miR-16 pre), restored luciferase activity to 70% of control cells transfected with vector followed by pre-miR-16 (vec + miR-16 pre). This data suggests that HuR can partially block miR-16 function.

### 2.5. HuR Does not Block miR-16 Repression of Endogenous Cyclin E1 mRNA

**Figure 5:**
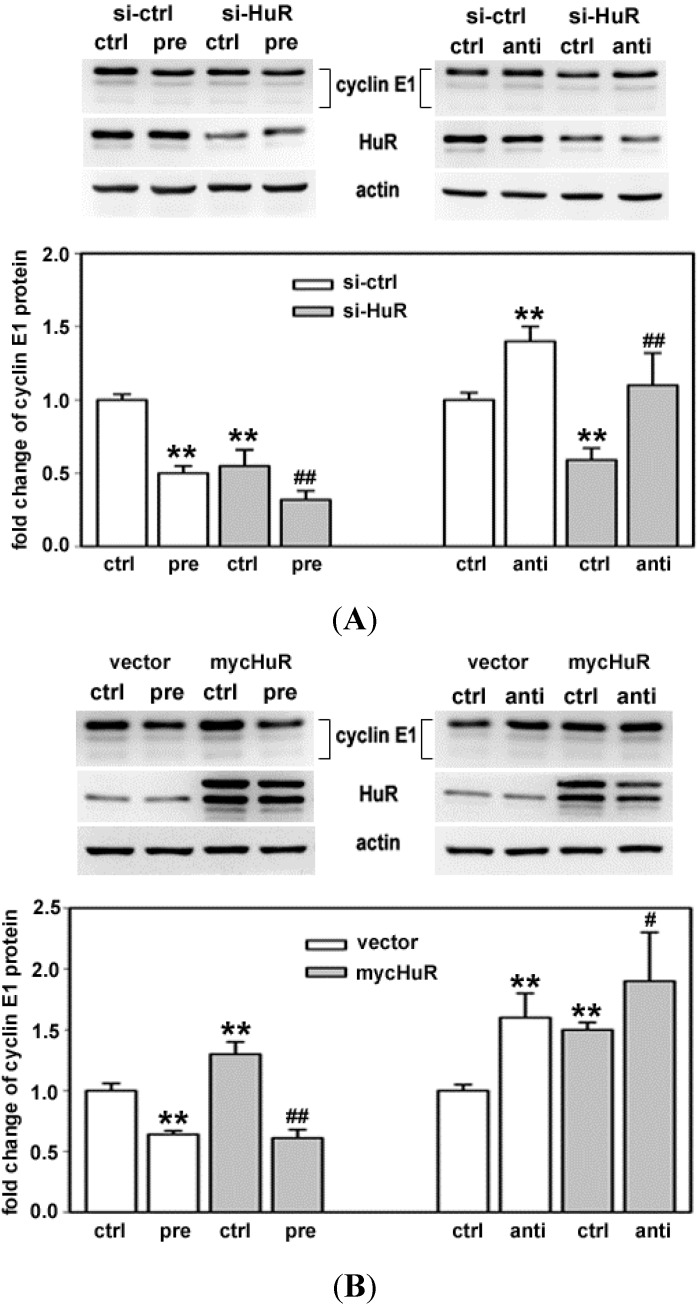
HuR and miR-16 coordinate cyclin E1 expression. MCF-7 cells were transfected with si-ctrl or si-HuR (**A**); pcDNA3.1 vector or pcDNA3.1mycHuR (**B**). 24 h after transfection, the cells were further transfected with control miRNA (ctrl), miR-16 precursor (pre) or antagomir (anti). 48 h after the additional transfection, the levels of cyclin E1 and HuR were detected by western blotting. The blots were reprobed with actin. Representative results are shown from three experiments. The data shown in the graphs are means of three independent experiments with standard deviation (** *p* < 0.01 *versus* si-ctrl or vec + ctrl miRNA; ^#^ *p* < 0.05 *versus* mycHuR + ctrl miR-16; ^##^ *p* < 0.01 *versus* si-ctrl or vec + ctrl miRNA.

To confirm that the changes seen with the reporter mRNA upon altering both HuR and miR-16 correspond to changes in endogenous cyclin E1, western analysis was performed in MCF-7 cells with altered levels of these post-transcriptional regulators. Figure 5A shows representative western blots (top) and the average fold change in cyclin E1 protein from three experiments performed in triplicate (bottom) in which cells were treated with control siRNA (si-ctrl) or siRNA targeting HuR (si-HuR) followed by pre-miR-16 (pre) or control miR (ctrl) treatment. Cyclin E1 level was lower in cells treated with HuR siRNA followed by pre-miR-16, as compared to cells treated with either alone (Figure 5A, left). The reduction in cyclin E1 protein resulting from si-HuR, was reversed by anti-miR-16 (Figure 5A, right).

Figure 5B shows representative western blots (top) and the fold change in cyclin E1 protein (bottom) in cells treated with vector (vec) or vector containing myc-HuR (mycHuR), followed by pre-miR-16 (pre) or control miR (ctrl) treatment. Overexpression of myc-tagged HuR, while increasing cyclin E1 level by itself, did not block miR-16 mediated decrease in cyclin E1 (Figure 5B, left). Consistent with this, anti-miR-16 further increased cyclin E1 protein in cells that were pretreated with myc-HuR (Figure 5B, right). Although the luciferase reporter data in Figure 4 suggests that myc-HuR can block miR-16 function in repressing cyclin E1, myc-HuR is not able to block the repressive effect of miR-16 on endogenous cyclin E1 mRNA.

### 2.6. Cyclin E1 mRNA Associates with Ago2

**Figure 6:**
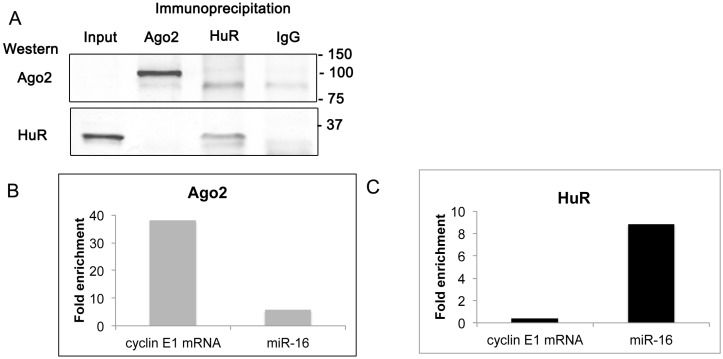
Cyclin E1 mRNA associates with Ago2. Immunoprecipitation of endogenous Ago2 or HuR was performed on untransfected MCF-7 cell extracts. (**A**) Western analysis for proteins precipitated with antibodies specific for Ago2, HuR, or nonspecific IgG. 40-μg of cell lysate was resolved in the Input lane. Blot was probed using anti-Ago2 and anti-HuR sequentially and imaged on a Li-Cor imager. Molecular weight markers are indicated to the right. The uncropped blot as well as a blot from a separate experiment is shown in Figure S1; (**B**) Immunoprecipitation with antibodies to Ago2 followed by qRT-PCR for cyclin E1 mRNA or miR-16. Expression was normalized to input and fold enrichment compared to immunoprecipitation with IgG is shown; and (**C**) Immunoprecipitation with antibodies to HuR followed by qRT-PCR for cyclin E1 mRNA or miR-16. Expression was normalized to input and fold enrichment compared to immunoprecipitation with IgG is shown. A representative experiment of three with similar results is shown.

To determine if endogenous HuR and miR-16 are both associated with the cyclin E1 3'UTR, we examined the association of both the miR-16 RISC complex and HuR with the cyclin E1 mRNA. Argonaute (Ago) proteins are integral components of the RISC complex, with Ago2 involved in cleaving target mRNAs. We used immunoprecipitation of HuR or Ago2 followed by either western analysis or qRT-PCR to interrogate this association. Figure 6A shows that upon immunoprecipitation of Ago2, Ago2 but not HuR was detected on a western blot. Likewise, after immunoprecipitation of HuR, HuR but not Ago2 was detected by western blotting. Although Ago2 was only faintly seen in the input lane (see Supplementary Figure S1), it was clearly present in the cell extract upon immunoprecipitation. A band, slightly lower in molecular weight than Ago2, was seen in both the HuR and IgG immunoprecipitates. These results indicate that a stable association between endogenous Ago2 and HuR could not be detected with this assay.

qRT-PCR analysis of RNA extracted from the immunoprecipitates showed that both cyclin E1 mRNA and miR-16 associated with Ago2 (Figure 6B), consistent with miR-16 regulation of cyclin E1 mRNA. miR-16 was also associated with HuR, (Figure 6C), while cyclin E1 mRNA association with HuR was much lower. These results show that both cyclin E1 and miR-16 can be found associated with Ago2, consistent with miR-16 RISC regulation of cyclin E1 mRNA. In contrast, only miR-16 was found associated with HuR. This could be explained by unstable association of HuR with the cyclin E1 mRNA under these assay conditions.

### 2.7. Discussion

Cyclin E1 overexpression is a prognostic factor for breast cancer [17,18], most recently being shown to be a strong prognostic factor for death in lymph node-negative breast cancer [45]. This study set out to further define defects in post-transcriptional regulation of cyclin E1 that result in its overexpression in breast cancer. We had previously shown that HuR upregulation contributes to cyclin E1 overexpression in breast cancer cell lines [28]. HuR itself is overexpressed in a subset of breast cancers, with its cytoplasmic localization correlating with poor prognosis [25,26,27,46]. In contrast to HuR, miR-16 destabilizes cyclin E1 mRNA [29,30] and is reduced in breast cancer [31,32]. Our current results show that HuR and miR-16 regulate cyclin E1 mRNA stability in concert in breast cancer cell lines. Overall, results suggest that miR-16 can override HuR upregulation of cyclin E1 without affecting HuR expression or directly affecting HuR association with the cyclin E1 mRNA.

HuR increases cyclin E1 mRNA stability and protein level by binding regions of the cyclin E1 mRNA 3'UTR that are U-rich, while miR-16 decreases cyclin E1 mRNA stability and protein level also dependent on cognate binding sites within the 3'UTR. Consistent with our results, PAR-CLIP analysis identified cyclin E1 as HuR target, with 2–4 U-rich elements in the cyclin E1 mRNA 3'UTR [34]. In addition, a separate PAR-CLIP analysis of HuR targets predicted overlap between HuR target sites and miR-16 family seed sequences [33]. Although the second miR-16 target site in the cyclin E1 3'UTR is in close proximity to a U-rich HuR binding site (8-nucleotides separate these sites), the sites do not overlap. miR-16 and HuR do not affect the other’s binding to the cyclin E1 3'UTR.

Interestingly, despite not affecting the other’s binding, overexpressed HuR partially blocks miR-16 repression of a reporter mRNA containing the cyclin E1 3'UTR, but does not block miR-16 repression of endogenous cyclin E1. This could be due to differences in HuR and/or miR-16 regulation of cyclin E1 mRNA within the context of the reporter *versus* the full length cyclin E1 mRNA. Differences in the RNA sequence outside of the 3'UTR in the endogenous and reporter RNAs very likely result in differences in RNA secondary structure. RNA secondary structure affects the accessibility of binding sites for RNA binding partners and thus their function [47,48]. In contrast, miR-16 blocks HuR-mediated upregulation of endogenous cyclin E1 (and the cyclin E1 3'UTR reporter). The mechanism does not appear to be via competing for or cooperating with HuR binding, as has been shown for other HuR/miRNA shared mRNA targets [36]. miR-16 repression of HuR-mediated cyclin E1 upregulation, could be due to a more stable association between miR-16 RISC with the cyclin E1 3'UTR, compared to a weaker HuR association with the cyclin E1 3'UTR, as seen by ribonucleoprotein immunoprecipitation (RNP-IP) and further discussed below.

Our results agree in part with a recent study showing co-regulation of a common target mRNA, COX-2, by HuR and miR-16 RISC [37]. In this study, overexpressed HuR similarly inhibited miR-16 targeting of a reporter mRNA containing the COX-2 3'UTR, but effects on endogenous COX-2 mRNA were not assessed. In addition, this study showed that miR-16 clearly associated with HuR and this association promoted the downregulation of miR-16. We similarly saw HuR and miR-16 association by RNP-IP (between endogenous HuR and miR-16), but no change in miR-16 expression upon HuR overexpression. We did not see appreciable association of the cyclin E1 mRNA with HuR by RNP-IP, although cyclin E1 mRNA clearly associated with Ago2. It is possible that association between endogenous HuR and cyclin E1 mRNA is unstable or below the level of detection in this assay without prior crosslinking.

Several miRNAs, including miR-16, have been shown to directly regulate HuR via interaction with its coding region or 3'UTR [35,36]. Our results clearly show that miR-16 did not indirectly affect cyclin E1 expression via direct regulation of HuR. We did not see an alteration in HuR protein by either overexpressing or inhibiting miR-16. Nor did HuR overexpression or knockdown change the level of miR-16. As discussed above, HuR was shown to influence the expression of COX-2 via reducing miR-16 [37], as well as to reduce miR-7, presumably by interfering with miRNA processing [33]. In the latter study, transcriptome-wide analysis of HuR targets by PAR-CLIP showed HuR interaction with many precursor or primary microRNAs as well as mature microRNAs [33]. The way in which HuR influences microRNA levels awaits further analyses.

In conclusion, we show that miR-16 and HuR co-regulate the cyclin E1 mRNA without influencing the other’s binding or expression. miR-16 regulation predominates, blocking upregulation of cyclin E1 by HuR. Our study focused on breast cancer cell lines, which have increased HuR and decreased miR-16, as compared to non-tumorigenic breast epithelial cell lines. Results suggest that both a decrease in miR-16 and an increase in HuR may be necessary for post-transcriptional overexpression of cyclin E1 in breast cancer. They also suggest that at least in some cases, HuR overexpression is not sufficient for upregulation of its targets in breast and perhaps other cancers. Further insight into the mechanisms of this co-regulation is necessary in order to determine if it can be exploited for future therapeutic intervention to decrease levels of cyclin E1.

## 3. Experimental Section

### 3.1. Cell Culture and Transfections

MCF10A, MCF-7, Hs578T, and SKBR3 cell lines from American Type Culture Collection (ATCC, Manassas, VA, USA) were cultured under conditions recommended by the manufacturer. Cells seeded onto 6 well plates were transfected using lipofectamine2000 (Invitrogen, Carlsbad, CA, USA) according to the manufacturer’s recommendations with one or more of the following, as indicated in the figure legends: miR-16 precursor, miR-16 antagomir, or negative control (40 nM; Ambion, Austin, TX, USA); Ago2 siRNA, HuR siRNA, or control siRNA (100 nM, Santa Cruz Biotechnology, Santa Cruz, CA, USA); pcDNA3.1 or pcDNA3.1 myc-HuR (4 μg) 24 h after transfection, total RNA was extracted for Northern blotting and real-time PCR. 48–72 h after transfection, protein was extracted for western blotting.

### 3.2. Constructs

pGEM-T Easy-cyclin E1 3'UTR and pGEM-T Easy-cyclin E1 CR378 were constructed as described previously [28]. Five separate pGEM-T Easy-cyclin E1 3'UTR segments (A–E) were generated by site-directed mutagenesis. The full-length cyclin E1 3'UTR was cloned into pMIR-REPORT luciferase construct (Ambion, Austin, TX, USA). Four or five residues in the miR-16 seed regions were mutated or the HuR binding sites were deleted by site-directed mutagenesis (Stratagene, La Jolla, CA, USA). All constructs were verified by sequencing. Primers are listed in Table 1.

**Table 1:** Primers for, generating cyclin E1 3'UTR segments A–E, 3'UTR mutations, and for qRT-PCR.

Names	Primers (from 5' to 3')
A (1–157)	Forward: GCAGTCTAGACCACCCCATCCTTCTCCACCA
	Reverse: CGACGATATCTGCCCTGTTTGATGCCATCCACA
B (135–291)	Forward: GCAGTCTAGATGTGGATGGCATCAAACAGGGCA
	Reverse: GCAGGATATCTGTTGTGGGAGTCCCTTAGGTCAA
C (229–385)	Forward: GCAGTCTAGAACCAGTGCGTGCTCCCGATG
	Reverse: CACCGATATCCCCGCAACCACCTGCTCCAC
D (323–445)	Forward: GCAGTCTAGAGGCGTGGCTCTCCTCGCAG
	Reverse: GAGACGGATATCCTGATAATGTGGAGAGGGCAGCCC
E (388–534)	Forward: GCAGTCTAGAAGCGTTGTGCAGAGCCCATAGC
	Reverse: GGACGATATCGTCTCAAAAACAGTATTATC
miR-16 site 1	Forward: ACACCAGTGCGTGCTCCCGAGACCTCGATGGAAGGTGCTACTTGACC
	Reverse: GGTCAAGTAGCACCTTCCATCGAGGTCTCGGGAGCACGCACTGGTGT
miR-16 site 2	Forward: GTGTACAATGCCTTTGATGAACTGTTTTGTAAGCGTTACGATATCTATCCATTTTTTAATAAAGATAATACTG
	Reverse: CAGTATTATCTTTATTAAAAAATGGATAGATATCGTAACGCTTACAAAACAGTTCATCAAAGGCATTGTACAC
HuR site 1	Forward: GATGGCATCAAACAGGGCAAAGTGGGTCAAGTAC
	Reverse: GTACTTGACCCACTTTGCCCTGTTTGATGCCATC
HuR site 2	Forward: GTGCTGCTATATCTATCCAAATAAAGATAATACTG
	Reverse: CAGTATTATCTTTATTTGGATAGATATAGCAGCAC
U6	Forward: CGCAAGGATGACACGCAAATTC
	Reverse: QuantiMir Universal Reverse primer
miR-16	Forward: TAGCAGCACGTAAATATTGGCG
	Reverse: QuantiMir Universal Reverse primer
Cyclin E1 mRNA	Forward: CGGCTCGCTCCAGGAA
	Reverse: TCATCTGGATCCTGCAAAAAAA
GAPDH mRNA	Forward: GGCCTCCAAGGAGTAAGACC
	Reverse: AGGGGTCTACATGGAAACTG

### 3.3. Northern Blotting and qRT-PCR

RNA was isolated using TRIzol, following the manufacturer’s instructions (Invitrogen by Life Sciences, Grand Island, NY, USA). Northern blotting and qRT-PCR were performed as described previously, with the same primer pairs, see Table 1 and [28]. RNA quality and concentration was assessed using a Nanodrop 2000 (Thermo Scientific, Waltham, MA, USA). For mRNA quantitation, cDNA was generated following the SuperScript II RT Protocol (Invitrogen). For miR-16 quantitation, a QuantimiR kit was used according to the manufacturer’s protocol (System Biosciences, Mountain View, CA, USA). qRT-PCR was performed following the SYBR Green PCR Master Mix protocol (Applied Biosystems, Foster City, CA, USA) on an Applied Biosystems 7500 Fast Real-Time PCR System. Threshold cycles (*C*_t_ values) were normalized to GAPDH for mRNA, and U6 for miR-16. For IP assays followed by qRT-PCR data were normalized to input cyclin E1 or miR-16 level and fold enrichment relative to IgG relative mRNA levels. For cyclin E1 3'UTR mRNA stability assays, cells were treated with actinomycin D (5 µg/mL) for the times indicated starting 24 h after transfection with miR-16 precursor or antagomir as previously described [28].

### 3.4. Western Blotting

Whole-cell lysates were resolved by SDS-PAGE, transferred to nitrocellulose membranes, blocked in 5% non-fat dry milk in TBS/0.05% Tween-20, and probed with antibodies specific for cyclin E1, HuR, Ago2 or β-actin (1:1000, Santa Cruz Biotechnology, Santa Cruz, CA, USA). Li-Cor Odyssey secondary antibodies and a Li-Cor imager were used for visualization and quantitation. Uncropped western blots from immunoprecipitation experiments can be seen in Figure S1.

### 3.5. Reporter Gene Assay

Cells of 50% confluence in 24-well plates were transfected using lipofectamine2000. MiR-16 precursor with equal amounts (200 ng) of pMIR-REPORT luciferase construct containing either wild type or mutant cyclin E1 3'UTR and pMIR-REPORT β-gal Control vector (for normalization) were co-transfected per well. Cell extracts were prepared 24 h after transfection, and luciferase activity was measured using the Dual-Light System (Applied Biosystems).

### 3.6. UV Cross-Link Competition Assays and Immunoprecipitation

pGEX-HuR plasmid (a kind gift of J. David Port, University of Colorado, Denver, CO, USA) was used to produce glutathione *S*-transferase (GST)-HuR in BL21DE3 pLysS *E. coli.* Bacterial lysate preparation and protein purification with Glutathione Sepharose 4 Fast Flow was performed as recommended by the manufacturer (GE Healthcare Bio-sciences, Pittsburgh, PA, USA). Protein concentrations were determined using Bio-Rad Protein Assay (Bio-Rad, Hercules, CA, USA). GST-HuR eluted as a doublet, with the upper band around 62 kDa and the faster migrating band at around 55 kDa (not shown). This faster migrating band is most likely a *C*-terminal cleavage product of the full length GST-HuR that lacks RRM3. Purified GST-HuR (50 ng) was incubated 20 min with ^32^P-labeled cyclin E1 3'UTR mRNA (Full length, FL) and A–E segments, Figure 4C) at room temperature. RNA-protein complexes were UV cross-linked on ice in a Stratalinker 1800 and subsequently resolved on a 10% SDS-polyacrylamide gel. Gels were dried and analyzed on a phosphorimager. For UV cross-link competition, GST-HuR was preincubated 15 min with 0 or 50 molar excess of unlabeled mRNA or 50 molar excess of unlabeled nonspecific competitor (cyclin E1CR378) before addition of labeled mRNA. For UV cross-link immunoprecipitation assays, MCF-7 cytoplasmic extracts (100 µg) were incubated overnight at 4 °C with mouse monoclonal HuR antibody. The immunocomplexes were incubated with 500 fmol ^32^P-labeled cyclin E1 3'UTR for 20 min at room temperature. RNA-protein complexes were UV-cross-linked and then precipitated by incubation with 20 µL agarose conjugate suspension (protein G-agarose, Santa Cruz Biotechnology) for 3 h at 4 °C with gentle rotation. The beads were washed, pelleted, and bound proteins eluted with SDS sample buffer before resolving on a 10% SDS-polyacrylamide gel.

### 3.7. Ribonucleoprotein Immunoprecipitation

Endogenous Ago2 and HuR were immunoprecipitated from MCF-7 cytoplasmic lysates as described [49]. Briefly, 400 μg of cell lysate was used per immunoprecipitation (IP). Cell lysates were precleared with 50% protein G-plus beads (Santa Cruz, SC-2002) in NT-2 buffer containing 5% BSA before adding one of the following: 15 μg of HuR antibody (mouse monoclonal 3A2, Santa Cruz Biotechnology, SC-5261), 5 μg Ago2 antibody (mouse monoclonal, Abcam, ab57113) or 5–15 μg IgG as a negative control (mouse, Santa Cruz, SC-2025). After a 4 h incubation at 4 °C ribonucleoprotein complexes were precipitated by adding 100 μL 50% protein G-plus beads and samples rotated overnight at 4 °C. After centrifugation, beads were washed 3X with NT-2 buffer and resuspended in 100 μL NT-2 buffer containing 20U RNAsin and 10U DNAse I (Promega, Madison, WI, USA) and incubated 15 min at 37 °C. Beads were once again washed and resuspended in 1 mL of NT-2 buffer. Then 900 μL of beads were centrifuged and resuspended in 100 μL of NT2 buffer containing 50 μg proteinase K and 0.1% SDS (30 min, 55 °C). After a brief centrifugation the supernatant was collected and combined with supernatant from a following wash with NT-2 buffer. RNA was extracted from the combined supernatants using TRIzol (Invitrogen by Life Technologies) and a Direct-zol RNA miniprep kit, according to the manufacturer’s protocol (Zymo Research, Irvine, CA, USA). qRT-PCR was performed for cyclin E1 mRNA and miR-16 on the resulting RNA. Protein sample buffer of 2× was added to the remaining 100 μL of beads to elute bound proteins, after centrifugation and supernatant removal.

### 3.8. Statistical Analysis

All experiments were repeated in triplicate at least three times. Data were presented as mean ± SD. Student’s *t* test was used to compare two groups (*p* < 0.05 was considered significant).

## Supplementary Materials

Supplementary materials can be found at http://www.mdpi.com/1422-0067/16/04/7112/s1.
