# Supplementary material for: MicroRNA-16 Modulates HuR Regulation of Cyclin E1 in Breast Cancer Cells
Source: Int J Mol Sci. 2015 Mar 30;16(4):7112–32. doi: 10.3390/ijms16047112 (PMC4425007; doi:10.3390/ijms16047112)
Supplement: Supplementary file 1 [file ijms-16-07112-s001.pdf]

Supplementary Information

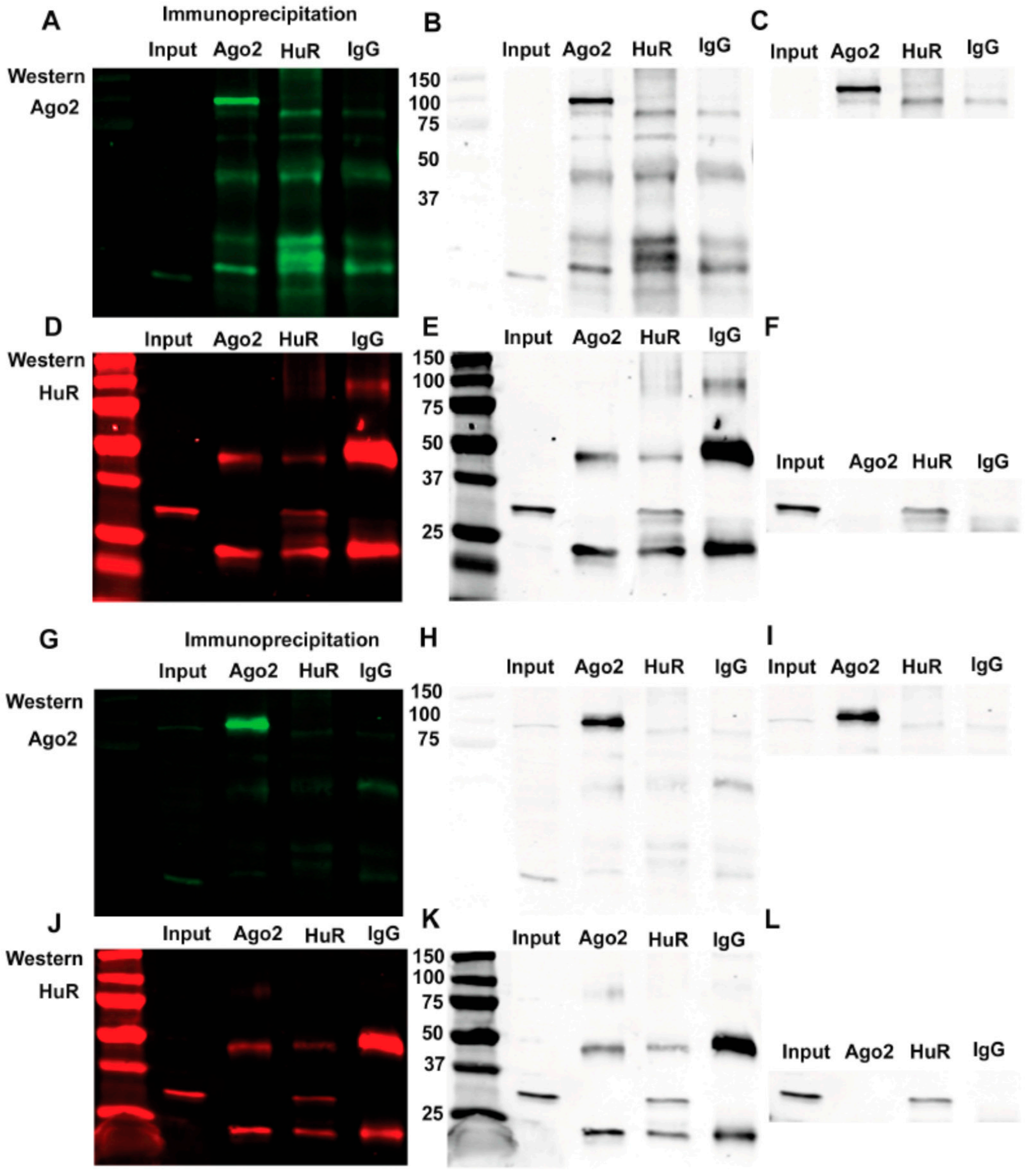

Figure S1. miR-16 associates with Ago2 but not with HuR. Immunoprecipitation of endogenous Ago2 or HuR was performed on untransfected MCF-7 cell extracts. 40-µg of cell lysate was resolved in the Input lane. Western analysis for input or proteins precipitated with antibodies specific for Ago2, HuR, or nonspecific IgG is shown. (**A**–**F**) Western blot for experiment shown in Figure 6; (**G**–**I**) western blot from a separate, replicate experiment. Blots were probed first with Ago2 antibody and an Odyssey IRDye 800CW-conjugated goat anti-rabbit secondary antibody (**A**,**G**), and then sequentially probed with HuR antibody and an Odyssey IRDye 680CW goat anti-mouse secondary antibody (**D**,**J**). Blots were imaged on an Odyssey Li-Cor imager and automatically pseudocolored. Molecular weight markers are in the left lane and seen in the 680 channel (pseudocolored red). Adobe Photoshop was used to invert the image, convert it to black and white, and auto level, as seen in (**B**,**E**,**H**,**K**). Images were cropped to show the Ago2 (**C**,**I**) or HuR (**F**,**L**) regions of the blots. Cropped images (**C**,**F**) are presented in Figure 6A. Bands seen at approximately 45 and 20 kDa are IgG heavy and light chains.
